# Plant-based therapeutics for leishmaniasis: A systematic review emphasizing human studies and clinical trial evidence

**DOI:** 10.1371/journal.pntd.0014389

**Published:** 2026-06-05

**Authors:** Alberta Serwah Anning, Vanesa Osmani, Stefanie J. Klug, Dorcas Obiri-Yeboah

**Affiliations:** 1 Department of Biomedical Sciences, School of Allied Health Sciences, College of Health and Allied Sciences, University of Cape Coast, Cape Coast, Ghana; 2 Chair of Epidemiology, TUM School of Medicine and Health, Technical University of Munich, Munich, Germany; 3 Department of Microbiology and Immunology, School of Medical Sciences, College of Health and Allied Sciences, University of Cape Coast, Cape Coast, Ghana; Food and Drug Administration, UNITED STATES OF AMERICA

## Abstract

**Background:**

Leishmaniasis is a parasitic disease caused by *Leishmania* species and transmitted through sand fly bites, affecting some of the most vulnerable populations globally. Current treatments are limited by high toxicity, poor tolerability, and resistance. Plant-based therapies offer a promising alternative, but human evidence has not been comprehensively reviewed. This review summarizes current evidence on the efficacy and safety of plant-based treatments for leishmaniasis in humans.

**Methodology/findings:**

We conducted a systematic review including studies that evaluated the efficacy and safety of plant-based treatments for leishmaniasis in humans. This review was registered with PROSPERO (ID: CRD42024567764). We searched PubMed, Scopus, and Web of Science from inception to May 28, 2024. Risk of bias was assessed using the Cochrane RoB 2 tool. We summarized the main study results qualitatively and quantitatively (where possible) by estimating risk ratios with 95% confidence intervals for treatment outcomes using the *meta* package in R. Ten studies met the inclusion criteria, nine from Iran and one from Sudan, all focused on cutaneous leishmaniasis (CL). Most used topical creams derived from medicinal plants, either alone or with conventional treatments. In studies combining herbal and standard treatments, four of six studies showed better outcomes in the intervention group. In studies using only the plant-based treatments compared to a standard treatment group, two of four showed better outcomes in the intervention group. Quantitative analysis of eight studies indicated significant healing improvements in the intervention group in three studies, specifically those using *Juniperus excelsa, Nigella sativa*, and poly-herbal formulations consisting of the pure extract mixture of *Althaea (A.) rosa*, *A. officinalis*, and members of the families Leguminosae, Faliaceae, Malvaceae, and Lythraceae (named Z-HE). Mild side effects such as itching and burning were reported with some herbal treatments, while conventional therapies caused more severe reactions in some cases. The risk of bias was mostly high in the studies.

**Conclusions:**

This review highlights the potential of certain plant-based compounds as adjunct or alternative therapies for CL. Despite promising results with *Plantago ovata, Juniperus excelsa*, and Z-HE poly-herbal extracts, current evidence is limited by methodological weaknesses. Larger, rigorously designed trials with broader representation are needed to confirm efficacy and safety and support policy integration in endemic regions.

## Author summary

Cutaneous leishmaniasis (CL) is a neglected disease that causes disfiguring skin sores, especially prevalent in tropical and subtropical regions. Existing treatments can be painful, expensive, and have serious side effects. Many people in affected areas turn to herbal medicines as an alternative, but it is not clear how well these treatments work in humans.

In this study, we systematically reviewed research on plant-based treatments for leishmaniasis in humans. We found ten studies, nine from Iran and one from Sudan. These studies mostly tested plant-based creams used either alone or in conjunction with standard drugs. Some combinations, like *Plantago ovata* with Glucantime or *Juniperus excelsa* with cryotherapy, showed improved healing rates. Most plant-based treatments caused only mild side effects, and some were better tolerated than standard treatments.

Although the results are encouraging, most of the studies had a high risk of bias. This review shows that plant-based treatments could help people with CL, especially in places where access to conventional treatment is limited. However, better quality research is needed before these alternatives can be recommended more widely.

## Introduction

Leishmaniases are a group of parasitic diseases caused by infection with any of the numerous species of *Leishmania* parasites, which are spread by the bites of infected sand flies [1]. The disease is endemic in about 90 countries globally, with tropical and subtropical countries most affected [1]. It is widely recognized as a neglected tropical disease (NTD), disproportionately affecting poor and marginalized populations in low and middle-income countries [2].

Clinically, leishmaniasis presents in three main forms: cutaneous leishmaniasis (CL), mucocutaneous leishmaniasis (MCL), and visceral leishmaniasis (VL) [3]. CL, the most common form, typically results in ulcerative skin lesions that may heal spontaneously but often leave disfiguring scars [4]. Though MCL is less prevalent, it causes severe damage to the mucosal tissues of the nose, mouth, and throat. VL, also known as Kala-azar, is the most severe form, affecting the internal organs, including the liver, spleen, and bone marrow. VL is fatal when not adequately treated [5].

The global burden of leishmaniasis remains substantial. An estimated 2–4 million incident cases occur each year, with approximately 70,000 deaths attributed to the disease annually [6]. CL alone accounts for 600,000–1 million new cases yearly [7]. Notably, around 70–75% of the CL cases are reported from ten countries, Afghanistan, Algeria, Brazil, Colombia, Costa Rica, Ethiopia, Iran, Peru, North Sudan, and Syria, where socio-political and environmental factors often exacerbate disease transmission and access to care [8]. VL has a clustered distribution across the globe, with over 90% of global cases reported from seven countries: Brazil, Ethiopia, India, Kenya, Somalia, South Sudan, and Sudan [9,10]. On the other hand, MCL is endemic to South America, particularly Brazil, Bolivia, and Peru [11]. Despite this burden, leishmaniasis continues to receive limited attention and funding relative to other global health priorities.

The *Leishmania* parasite exists in two morphological forms: the promastigote, which develops in the sand fly vector, and the amastigote, which multiplies within human host macrophages following transmission [12]. It is this intracellular replication of amastigotes that underlies the pathology of the disease.

Chemotherapeutics used in treating leishmaniasis range from Aminoglycoside Paromomycin, Amphotericin B, the Pentamidinols, Alkyl phosphocholine miltefosine, and Pentamidine [13]. Despite these treatment alternatives, the drugs have high toxicity, life-threatening side effects, high cost, long and painful treatment, and contribute to the emergence of parasite resistance [14].

Additionally, most endemic countries have limited drug choices. Some drugs are included on national essential medicines lists, but stockouts, high costs, and registration issues frequently limit consistent access [15]. Meglumine antimoniate (commercially known as Glucantime), although the least expensive drug available for the treatment of leishmaniasis, costs between $125.74 and $418.52, an amount that remains prohibitively high for the majority of affected individuals, most of whom are economically disadvantaged [16,17]. Aside from the costs, this drug requires parenteral administration and has more toxicity concerns as well. In addition, the emergence of parasite resistance further limits their long-term efficacy [7,8].

The lack of available vaccines, coupled with the shortcomings of chemotherapy, has necessitated using natural products as important alternatives for leishmaniasis [18]. Many people affected by this NTD frequently turn to natural remedies such as plant parts and oils as affordable and conveniently accessible alternatives to commercial antileishmanial drugs. The high cost, limited availability, and side effects associated with conventional treatments drive this preference among resource-limited populations [19,20].

Many medicinal plants produce secondary metabolites such as alkaloids, flavonoids, and terpenoids that exhibit antiparasitic activity [21,22]. Though the promise has been indicated by plant compounds, many of the studies examining their activity against *Leishmania* have predominantly remained at the *in vitro* level [23,24]. Such studies generally explore the direct cytotoxicity of plant extracts or isolated compounds against the promastigote and amastigote stages of the parasite [24–26]. Although *in vitro* results are useful preliminary data, *in vivo* efficacy is not always mirrored by them, owing to issues related to bioavailability, metabolic interaction, and host immune reaction [21].

While several others have extensively examined the efficacy of plant-based compounds (e.g., essential oils, garlic extracts) against leishmaniasis *in vitro* and in mice, highlighting promising results in reducing parasitic load and lesion volume [23–30], no review of the evidence is available on the effects of such alternatives on humans. This systematic review aims to provide a comprehensive overview of the available literature on plant-based treatments used in the treatment of leishmaniasis in humans, evaluating their efficacy and safety profiles.

## Methods

The study protocol for this systematic review has been registered in the International Prospective Register of Systematic Reviews (PROSPERO ID CRD42024567764). The work was conducted following the PRISMA guidelines [31].

### Eligibility criteria and search strategy

The study eligibility criteria were based on the PICO (population, intervention, comparators, and outcome) scheme. The population of interest (P) was children and adults who had been clinically diagnosed with any form of leishmaniasis. The treatment or intervention of interest (I) was the application of plant-based compounds, either used as monotherapy or as an adjunct treatment with any of the conventional treatment options (e.g., antimonials, amphotericin B, miltefosine). Studies were included if they had a comparison group, including standard/conventional treatment or placebo (C). The outcomes of interest (O) included efficacy (cure/healing rate, parasitological clearance/reduction in parasitemia) and safety outcomes (adverse events, toxicity). We included non-randomized trials and randomized controlled trials (RCTs). We did not include observational studies, review articles, meta-analyses, case studies, editorials, book chapters, or research articles focused solely on *in vivo* and *in vitro* activities. There were no restrictions based on language.

We systematically searched the databases PubMed, Web of Science, and Scopus, and the studies were considered from the inception of these databases until 28th May 2024. The complete search strategy used in PubMed is detailed in S1 Table of the appendix. We combined terms related to leishmaniasis, herbal medicine, plant-based compounds, and treatment outcomes in order to find relevant studies. The search strategy was adapted for the Web of Science and Scopus. Additionally, we hand-searched in Google Scholar using search terms such as “plant-based” and “leishmaniasis”.

All search results were downloaded and managed in EndNote X9 (Clarivate Analytics, Philadelphia, PA, USA), where duplicates were removed. The title/abstract and full-text screening were done using Rayyan (Rayyan Systems Inc., Doha, Qatar). Screening for eligibility was done independently by two authors (ASA, VO), results were compared, and where there were disagreements, discussions were held until a final decision was made.

### Data extraction and risk of bias

We extracted data using a standardized extraction sheet developed in Microsoft Excel (Microsoft Corporation, Redmond, WA, USA). The following information was extracted from each included study: publication details (first author and year), study design, year of study conduct, world region (continent), subregion and country, city, recruitment setting, sample sizes for intervention and control groups, age of participants in both intervention and control groups, number of female and male participants, descriptions of the intervention and control conditions, type of plant used, its preparation and administration, duration of the intervention, type of leishmaniasis, outcome definitions, and study results regarding efficacy and side effects. VO and ASA extracted the data independently and discussed any discrepancies before resolution.

The methodological quality of the papers was evaluated using the Cochrane Collaboration’s Risk of Bias tool (Rob 2.0) [32]. The risk of bias assessment involved five domains: bias from the randomization process, bias due to deviations from intended interventions, bias resulting from missing outcome data, bias in outcome measurement, and bias in the selection of reported results. Each study was then classified as having either a high risk, some concerns of bias, or a low risk of bias in these domains. The risk of bias assessment was conducted by evaluating the ‘intention-to-treat’ effect, with efficacy (cure/healing rate or parasitological clearance/reduction) as the primary outcome of interest. An overall risk of bias was assigned to each study based on the ratings from all domains. VO and ASA independently assessed the risk of bias and clarified any discrepancies in the evaluation. The risk of bias results are reported visually, considering results on all five domains of ROB-2 as well as the overall assessment.

### Data synthesis and analysis

We summarized the search results using a PRISMA flowchart and the main study characteristics in a tabular format. The proportion of female participants was determined using the total number of individuals enrolled in the studies as the denominator. The study design is reported as assessed by both reviewers and not necessarily as described in the included studies. If randomization was unclear, the studies were considered as non-randomized trials.

For studies that reported overall population numbers and the number of participants in both experimental and control groups, which achieved complete healing or improvement (defined as complete or almost complete healing or re-epithelialization of the lesions), or these numbers could be calculated, we estimated risk ratios to assess the intervention effects for all studies included in the analysis. The risk ratios and the corresponding 95% confidence intervals (CIs) of individual studies are presented in a forest plot without a pooled estimate. The statistical analysis was conducted using the add-on package meta in R version 4.4.1.

## Results

### Search results

**Fig 1:**
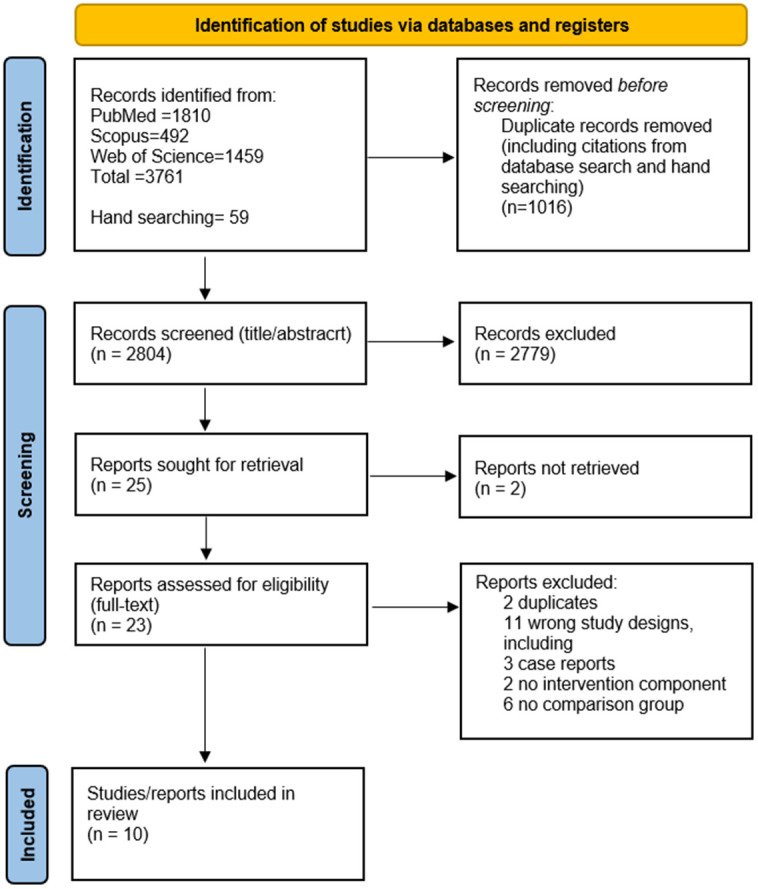
Modified PRISMA flowchart of the study selection process.

In total, there were 3761 articles retrieved from the three databases, PubMed, Scopus, and Web of Science, whereas 59 were obtained by hand-searching in Google Scholar (Fig 1). After removing 1016 duplicates, 2804 records were included in the title and abstract screening. Following this, 25 publications were sought for retrieval, out of which two could not be retrieved, even after contacting the corresponding authors [33,34]. Full-text screening was performed with 23 articles, of which 10 met the eligibility criteria and were therefore included in the qualitative synthesis of this systematic review [35–43] Thirteen reports were excluded: two due to duplication and eleven due to inappropriate study designs (three case reports, two lacking intervention components, and six lacking comparison groups), as specified in the exclusion criteria.

### Study characteristics

The main study characteristics are presented in Table 1. Out of the ten included studies, 9 (90%) [35,37–44], were conducted in Iran and one in Sudan [36]. All ten studies reported individuals diagnosed with CL and were published from 1999 to 2023. No studies included participants diagnosed with VL and MCL. Nine [35,37–40,42–44] of the ten studies were randomized controlled trials (RCTs) [41], and one was considered a non-randomized trial [36]. All studies recruited participants from either clinics/hospitals or research centers. Nine [36–44] out of 10 studies reported on the mean age of participants for both intervention and control groups, which ranged between 18.5 and 42.1 years. The remaining study included participants as young as 10 months to 69 years old. The majority of the studies included more male than female participants. Most studies used different plant-based creams, which were usually applied twice a day topically among participants diagnosed with CL. A variety of plants were used.

**Table 1:** Main characteristics of the included studies (n = 10).

Study	Country/ Region	Years of study conduct	Study design	Recruitment setting	Age of participants (I/C) in years	Female (%)	Plant used	Preparation and usage
Aghaei et al. (2018)	Iran/Asia	2017	RCT	Health centre	Mean, SD 35.53 ± 13.22/34.26 ± 13.80	30.0	Olive oil	To produce 150 g of olive oil, 2 kg of pomace olive and 500–600 cc of petroleum solvent were extracted using a Soxhlet for 3–4 hours. The solvent was then removed via condensation for 2–3 hours, yielding pure oil. Oxygen gas with 200 ppm ozone was bubbled through the oil at 1.0 L/min for 3 weeks to create ozonated olive oil with a Vaseline-like texture and an ozone scent. Topically; Twice daily
Ebrahimzadeh et al. (2022)	Iran/Asia	2017-2019	RCT	Clinical centre	Mean, SD 27.44 ± 17.37/26.94 ± 19.28	36.8	*Sambucus ebulus*	The fruit of *S. ebulus* was gathered from rural regions of Sari (Mazandaran Province, Iran), dried, and ground into powder at room temperature in the absence of light. The extract was then obtained by soaking the powdered fruit in methanol for three days. Topically; Twice daily
Ebrahimzadeh Ardakani et al. (2023)	Iran/Asia	2017-2018	RCT	Research centre and clinic	Mean, SD 25.00 ± 17.56/23.84 ± 13.49	59.5	*Plantago ovata*	Equal parts of ground *P. ovata* powder and vinegar were combined and left to sit for approximately 48 hours until the mixture reached a suitable consistency. Used topically; Twice daily
Gholami et al. (2000)	Iran/Asia	1997-1998	RCT	Research centre	Mean 18.5/ 23.7	48.0	Garlic	A cream was formulated using a Mantis base containing 5% garlic extract. Topically: Twice daily
Jaffary et al. (2014)	Iran/Asia	2009-2010	RCT	Research/ medical centre	Mean, SD 25.8 ± 3.7/ 24.6 ± 4.2	25.0	*Achillea millefolium*	Carbomer 934 was dispersed in distilled water and neutralized with triethanolamine to enhance its consistency. Dried *A. millefolium* was mixed into a solution containing 20% glycerin and 5% propylene glycol. To improve solubility, 2% parabens and 100 g of water were then added to the mixture. Topically; Twice daily
Jaffary et al. (2014)	Iran/ Asia	NR	RCT	Research centre	Mean, SD 20.6 ± 12.4 vs. 19.8 ± 11.5 vs. 22.9 ± 13.6	46.1	*Cassia Fistula*	Five hundred grams of *C. fistula* powder was mixed with 70% ethanol at a 1:3 ratio and placed in a percolator. After 48 hours, the extract was collected and concentrated under vacuum distillation. Another 500 g batch was mixed with water at the same ratio, boiled for 30 minutes, and similarly concentrated by vacuum distillation. Gauze soaked in the extract was applied to the lesions once daily.
Khalid et al. (2004)	Sudan/ Africa	1999-2000	Non-RCT	Teaching hospital	Mean overall 28.2	47.2	Multiple: Leshmanol, neem (*Azadirachta* *indica)*, garad (*Acacia nilotica)*, garlic	Unclear Majorly topically twice a day
Nilforoushzadeh et al. (2010)	Iran/ Asia	2006-2007	RCT	Research centre	Mean, SD 20.81 ± 12.26/ 21.42 ± 11.46	28.7	*Nigella sativa*	*N. sativa* seeds were purchased from Pakan Seed Company in Isfahan, and a 60% hydroalcoholic extract was prepared by the pharmacognosy department. Gauze soaked with 60% honey-based *N. sativa* extract was applied and replaced every 12 hours.
Parvizi et al. (2017)	Asia/Iran	2015-2016	RCT	Medical centre	Mean, SD 38.91 ± 13.49/ 42.10 ± 14.54	40.3	*Juniperus excelsa*	The hydroalcoholic extract of the leaf was obtained using a percolator apparatus. The resulting extract was then concentrated and dried. Both the *J. excelsa* extract (JE) and the placebo were formulated as oil-in-water creams. Topically; Thrice daily
Zerehsaz et al. (1999)	Iran/Asia	NR	RCT	Medical centre	Range, 10 months -65 years/ 1.5-69 years	50.9	multiple: *Althaea rosa, Althaea officinalis*, plants members of the families Leguminosae,Faliaceae, Malvaceae, and Lythraceae	Topical herbal extract Z-HE prepared as a black paste Applied the paste for five consecutive days

I, Intervention; C, Control; RCT, randomized control trial; SD, standard deviation; NR, not reported; Z-HE, A topical herbal extract consisting of a pure extract mixture of *Althaea rosa, Althaea officinalis*, and members of the families Leguminosae, Faliaceae, Malvaceae, and Lythraceae.

### Risk of bias

**Fig 2:**
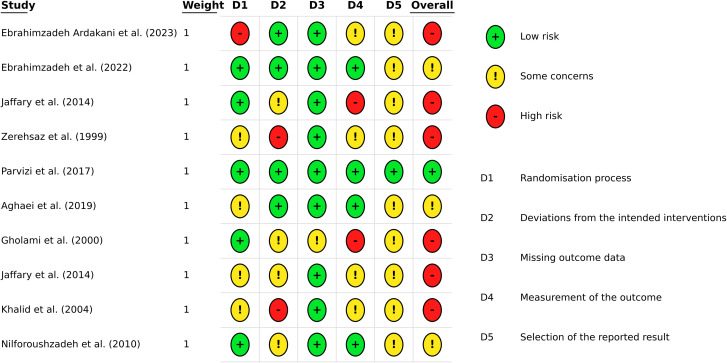
Risk of bias (ROB-2) results of included studies (n = 10).

Figs 2 and S1 present the risk of bias results for the 10 studies across various domains, followed by an overall evaluation. One study [42] had a low risk of bias, 3 [38, 39, 41] were assessed with some concerns overall, and the remaining six studies [36,37,40,41,43,44] had a high risk of bias. The studies were judged to have a high risk of bias due to the absence of pre-specified analysis protocols, which raises concerns about selective or data-driven analyses. Additional sources of bias included a lack of clarity regarding multiple outcome measurements and, in some cases, issues with randomization, blinding, and outcome assessment.

### The effect of plant-based creams/ointments on the treatment of leishmaniasis

Overall, most studies (60%) reported statistically significant clinical improvements in the intervention group receiving a variety of plant-based creams/ointments alone or with a standard treatment such as Glucantime (Table 2). Among the six [35,38,39,41–43] studies where the intervention group received both the plant-based cream and the standard treatment or cryotherapy, and the control group received only the standard treatment or cryotherapy, four found significant improvements in the intervention group, and two found no differences between the groups. In the remaining four studies [36,37,40,43], where the plant-based cream group was compared with groups receiving either Glucantime, Pentostam, or placebo, only two found significant improvements in the intervention group in comparison to the control group. Specifically, one [36] found significant improvements when using multiple: Leshmanol, Neem, Garad, and Garlic in comparison to the intravenous Pentostam group. The other study [40] compared the usage of Z-HE poly-herbal with Glucantime. Out of six studies [35,37,39,40,42,44] that reported side effects, three reported mild reactions to the plant-based creams, including burning, irritation, and itching, while one study reported urticarial and generalized pruritus in the control group receiving Glucantime.

**Table 2:** The interventions used and the main findings on the effects of plant-based creams/ointments on the treatment of leishmaniasis.

Study	Intervention	Comparison	Duration of intervention	Intervention n	Control n	Outcome definition	Main findings	Included in the quantitative synthesis
Aghaei et al., 2018	Topical ozone saturated olive oil and Glucantime	Glucantime	8 weeks	15	15	Decrease in lesion size Side effects	Statistically significant improvement in the intervention group Only transient burning sensation in some patients, otherwise well tolerated	No
Ebrahimzadeh et al. (2022)	5% paraffin and vaseline-based *S. ebulus* ointment and standard treatment	Placebo and standard treatment	Until complete improvement or 12 weeks	45	50	Complete re-epithelialization at day 42	No differences between the groups	Yes
Ebrahimzadeh Ardakani et al. (2023)	*P. ovata*, vinegar, and Glucantime applied topically	Placebo and Glucantime injection	Until complete improvement or 8 weeks	21	21	Complete improvement (100%) as in the percentage of lesions improved Mean lesion area in cm	Statistically significant improvement in the intervention group Statistically significant improvement in the intervention group only at 8 and 12 weeks	Yes^*^
Gholami et al. 2000	Topical 5% garlic cream	Placebo cream	40 days	96	75	Complete disappearance of the ulcer and the absence of parasites in the smear	No differences between the groups	Yes
Jaffary et al. (2014)	5% yarrow (containing 5% polyphenol) and Glucantime	Placebo and Glucantime	Up to 12 weeks	30	30	Complete or partial cure rate Side effects	No differences between the groups Mild to moderate itching and severe itching in 8 and 1 participant in the intervention group, respectively	Yes
Jaffary et al. 2014	Extract-soaked gauze of Concentrated boiled and hydroalcoholic extracts of *C. fistula*	Intralesional injection of Meglumine Antimoniate	Until healing or up to 4 weeks	Gr 1: 55 Gr 2: 55	55	Complete disappearance of the ulcer and the absence of parasites in the smear	Statistically significant improvement in the control group	Yes
Khalid et al. 2004	Topical Leishmanol, methanol extracts of neem, garad, and garlic	Intravenous Pentostam	8 weeks	NR	NR	Complete healing defined as a smooth scar and no detectable parasites	Only the intervention group receiving garad showed statistically significant improvements	No
Nilforoushzadeh et al. 2010	Intralesional Glucantime along with topical honey-based hydroalcoholic extract *N. sativa* 60%	Topical honey and intralesional Glucantime	12 weeks	75	75	Complete re-epithelialization of cutaneous lesions Side effects	Statistically significant improvement in the intervention group In the end of follow-up, only about 7% of the intervention showed burning, itching, inflammation an allergic reaction	Yes
Parvizi et al., 2017	5% hydroalcoholic extract of leaves of *J. excelsa* as a topical cream and cryotherapy	Placebo and cryotherapy	Up to 12 weeks	33	29	Complete cure (re-epithelization at >90% and decrease in lesion size) Side effects	Statistically significant improvement in the intervention group Redness and itching after 5 weeks of JE usage	Yes
Zerehsaz et al. (1999)	Topical herbal extracts consisting of a pure extract mixture of *A. rosa, A. officinalis*, and members of the families Leguminosae, Faliaceae, Malvaceae, and Lythraceae (named Z-HE) and sterile normal saline injection	Topical placebo and Glucantime	5 days; measurements after 6 weeks	86	85	Complete or partial cure rate Side effects	Statistically significant improvement in the intervention group Urticaria and generalized Pruritus only in the control group	Yes

*Only the first outcome was considered for the quantitative synthesis; n, number of participants; NR, not reported.

### Risk ratios of treatment effects

**Fig 3:**
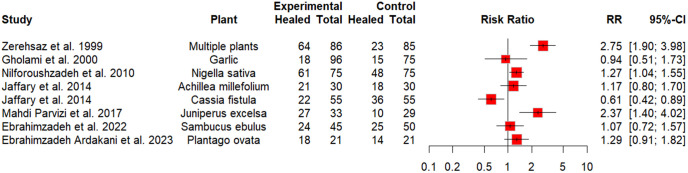
The effects of plant-based creams on the healing rate of cutaneous leishmaniasis. RR, risk ratio; CI, confidence interval.

Based on our analysis (Fig 3), including eight studies, we see significant differences in the healing rates among the individuals receiving the plant-based creams compared to the placebo group, only in half of the studies (n = 4). Three of these studies found significantly higher healing rates among those receiving a cream based on multiple plants (Leshmanol, Neem, Garad, Garlic) [40] [RR 2.75 (95%CI 1.90-3.98)], *J. excelsa* [42] [RR 2.37 (95%CI 1.40-4.02)] and *N. sativa* [35] [RR 1.27 (95%CI 1.04-1.55)], respectively, in comparison to the control groups. One study found a 39% lower probability of healing among *C. fistula* cream users compared with controls who received an intralesional injection of meglumine antimoniate [RR 0.61 (95%CI 0.42-0.89)]. For the remaining studies, no significant differences were found between the plant-based cream group and the control group.

## Discussion

Our study aimed to summarize the use of plant-based medicines in the treatment of leishmaniasis in humans worldwide. Most included studies were conducted in Iran [35,37–44], indicating a regional focus and limited geographic diversity, with only one study from Sudan, Africa [36]. This concentration underscores the need for further research in underrepresented endemic regions such as Africa and South America, where ethnobotanical knowledge is rich but clinical validation is scarce.

Out of six trials using plant-based therapies alongside standard treatments such as Glucantime or cryotherapy, four demonstrated increased efficacy in treating leishmaniasis compared to standard treatment alone. Plants such as *P. ovata, S. ebulus*, *A. millefolium,* and *J. excelsa* showed positive results as adjunct topical treatments. These formulations showed enhanced lesion re-epithelialization and higher cure rates, indicating potential synergistic interactions between plant compounds and antileishmanial drugs. *P. ovata*, known for its diverse pharmacological activities [45] yielded an 85.7% cure rate when combined with Glucantime, surpassing the 66.7% rate of Glucantime alone. *J. excelsa*, with known antimicrobial, respiratory, cardiovascular, and anti-inflammatory properties [46–48] showed promise as a topical adjuvant, especially when paired with cryotherapy. Its bioactivity is attributed to phytochemicals like flavonoids, saponins, and terpenes that induce parasite apoptosis, disrupt cell membranes, and generate reactive oxygen species (ROS) [49], enhancing parasite clearance and immune responses. Cryotherapy is commonly employed in the management of CL, particularly for uncomplicated lesions that exhibit minimal lymphocutaneous involvement, are of less than three months’ duration, and are limited in number (fewer than four), as well as in patients who are not candidates for systemic therapy [50–52].

The Z-HE poly-herbal extract, composed of *A. rosa, A. officinalis*, and members of the Leguminosae, Faliaceae, Malvaceae, and Lythraceae families, also showed a high complete healing rate with no reported side effects. While this activity is promising, the attribution of the efficacy of the exact ingredient is unclear. *A. officinalis* is well-known for its antioxidant and wound-healing properties [53], whereas information on *A. rosa* and other associated families is limited. Other treatments with mixed outcomes included *C. fistula* extracts, which showed moderate cure rates (40% for boiled, 36.4% for hydroalcoholic) compared to 65.5% with intralesional meglumine antimoniate [43]. Though this effect was inferior in comparison to the standard treatment, there are other reports of its usefulness in mild cases or as an adjunct [48,54]. *A. millefolium* (Yarrow) gel combined with Glucantime resulted in a 70% cure rate vs. 60% in the control group. While the difference was not statistically significant, the additive effects of *A. millefolium* are possible [55,56].

Glucantime was the most commonly used standard drug, featured in 7 out of 10 studies either as monotherapy or as part of combination therapy [35,38,39,41,44] or as the comparator [40,44]. However, it is associated with painful administration, systemic reactions, and known organ toxicities [57–59] Topical plant-based treatments used in the included studies generally reported mild side effects, such as localized redness, itching, and irritation, which were most notable with *J. excelsa* [42], *N. sativa* [35], and *A. millefolium* [44]. The Z-HE poly-herbal group reported no side effects, while the control group using Glucantime experienced urticaria and generalized pruritus, suggesting better tolerability of some herbal alternatives [40]. *P. ovata* is largely considered safe, although it may interact with co-administered drugs [60,61]. While this systematic review compared plant-derived compounds with Glucantime, Pentostam, and cryotherapy, other chemotherapeutic agents that are commonly used for the treatment of leishmaniasis include Paromomycin, Amphotericin B, Miltefosine, and Pentamidine, which have shown different clinical effectiveness for the treatment of the various forms of leishmaniasis [7,62]. Since the reviewed studies did not examine plant-derived compounds in comparison with the aforementioned chemotherapeutic agents, the effectiveness of plant-derived compounds in comparison with all the chemotherapeutic agents for the treatment of leishmaniasis may be questionable, and further studies would be needed for a better and broader comparative study of the therapeutic potential of plant-derived compounds.

Nevertheless, our findings complement and extend previous reviews, which have highlighted the potential of natural products, especially flavonoids, saponins, and terpenes, in *in vitro* and experimental models [23–30]. While those studies emphasized preclinical pharmacology, our review synthesizes limited but valuable human clinical evidence, bridging the gap between laboratory and real-world treatments. Odonne and colleagues documented the ethnopharmacological diversity of Amazonian antileishmanial plants, but noted a lack of translation into clinical evaluation, a gap which is also evident here, as South America remains underrepresented [63]. A closer examination of the more effective plant-based interventions in this review suggests that several share common classes of secondary metabolites, particularly flavonoids, terpenoids, and saponins. Notably, plant candidates demonstrating higher cure rates or improved lesion healing, such as *J. excelsa, P. ovata*, and *A. millefolium*, are known to contain these bioactive compounds, suggesting that their clinical benefits may be driven by shared phytochemical properties rather than isolated plant effects. Evidence from preclinical and *in vitro* studies supports this observation. For instance, flavonoids such as quercetin have been shown to inhibit *Leishmania* parasite proliferation, induce reactive ROS-mediated cell death, and modulate host immune responses [64]. Similarly, terpenoids have demonstrated the ability to disrupt parasite cell membranes, induce mitochondrial dysfunction, and trigger apoptosis through oxidative stress pathways [24,65]. In addition, flavonoid derivatives have been reported to inhibit key parasite enzymes, including cysteine proteases essential for parasite survival and replication [66]. Collectively, these mechanisms provide a plausible biological basis for the improved outcomes observed in adjunct plant-based treatments and highlight the importance of identifying, isolating, and standardizing active compounds in future clinical development.

The underrepresented regions endure high leishmaniasis prevalence fueled by poverty, limited healthcare infrastructure, and environmental and socio-economic vulnerabilities, underscoring the urgent need for region-specific clinical validation of traditional medicines. Other authors also noted that combination strategies may improve outcomes, which aligns with our findings, where adjunct therapies such as *P. ovata* or *J. excelsa* enhanced cure rates compared to standard therapy alone [25,29,58]. Methodological shortcomings, such as small sample sizes, variable outcome measures, and limited reporting of adverse events, have been repeatedly flagged in plant-based leishmaniasis studies [57,58,63]. and our review identified similar limitations.

Other researchers reviewing natural products with anti-leishmanial potential have stressed the importance of rigorous pharmacological evaluation before clinical translation [23–30], reinforcing that promising plant-derived therapies remain under-validated in human trials. Oliveira and colleagues emphasized the diversity of marine and plant-derived compounds used against protozoan diseases, including leishmaniasis, illustrating the vast reservoir of natural bioactives yet to be clinically exploited [67].

Despite the abundance of conventional VL trials, plant-based clinical research remains scarce. Closing this gap requires disease-specific trial designs and infrastructure tailored for natural products. Adaptive, multi-arm, multi-stage trials can evaluate plant extracts alongside standard therapies, such as miltefosine combinations. Outcomes should be stratified by species (*L. donovani vs. L. tropica*), supported by preclinical studies using hamster models and nanoformulated extracts to optimize systemic pharmacokinetics for spleen and liver penetration [68,69]. Decentralized rural trial sites, PCR-based parasite load biomarkers, and community health worker engagement can help overcome logistical barriers to testing plant-based compounds in VL [70]. Groups like Drugs for Neglected Diseases initiative (DNDi) and LeishRIV can help move this work forward by offering targeted funding and creating faster regulatory routes. A practical next step would be to start with pilot Phase 1 studies using standardized nanoformulated plant-derived extracts.

### Strengths and limitations

To our knowledge, this is the first systematic review that summarizes the evidence on the potential of plant-based alternatives in the treatment of leishmaniasis in humans. We comprehensively summarized the evidence while also assessing the risk of bias of included trials using the Cochrane ROB-2. In addition, we calculated risk ratios to summarize the results quantitatively by estimating standardized effect sizes to facilitate comparison across trials.

However, based on the Cochrane ROB-2 tool, most trials had a high risk of bias [36,37,40,41,43,44] with limitations such as poorly described blinding and randomization, inconsistent outcome definitions, short follow-up durations, and inadequate adverse effect reporting. Only one trial was assigned a low risk of bias [42]. Additionally, several trials lacked clear statements on ethical approval, informed consent, and allocation concealment, raising concerns of compliance with international standards. No pooled estimates could be calculated because there was heterogeneity in the investigated plants.

A key limitation of this review is that, although designed to include studies across all clinical forms of leishmaniasis, only investigations focused on CL met the inclusion criteria. This reflects the relative scarcity of plant-based therapeutic studies targeting VL, which is systemic, affecting the spleen, liver, and bone marrow, and associated with substantial morbidity and mortality [8,9]. Because visceral leishmaniasis is a complex disease, it usually requires systemic treatment along with careful assessment of how the drug behaves in the body, where it distributes in tissues, and its potential toxicity [71]. Additionally, studies involving viscerotropic species typically require more sophisticated *in vivo* models and advanced laboratory infrastructure due to distinct host-pathogen interactions and immune responses [72]. These scientific and logistical challenges likely contribute to the paucity of clinical or early-phase VL studies compared to those for CL.

## Conclusion

There is limited evidence that supports the potential of selected plant-based treatments, especially in combination with conventional therapies, for improved outcomes in CL. While results for *P. ovata, J. excelsa*, and Z-HE poly-herbal are promising, further large-scale, standardized clinical trials with ethical rigor and comprehensive adverse effect reporting are essential. Also, more studies are needed, particularly in Africa and Latin America, to validate these therapies and fully realize their potential in global leishmaniasis control. The development of affordable, well-tolerated herbal therapies remains not just a scientific opportunity but an ethical imperative for endemic, resource-limited regions to manage the NTD, leishmaniasis. Integrative public health approaches that combine validated herbal adjuncts with standard care, enhanced disease surveillance, and community engagement can ultimately improve treatment outcomes, reduce adverse effects, and advance control programs in neglected populations globally.

## Supporting information

S1 Table PubMed search strategy for studies on plant-based therapeutics for leishmaniasis emphasizing human studies and clinical trial evidence: A systematic review. (DOCX)

S1 Fig The results of the risk of bias (ROB-2) assessment by the evaluated domain. (DOCX)

S1 PRISMA 2020 Checklist licensed under CC BY 4.0. Original source: Matthew J. Page et al. (2021). PRISMA 2020 explanation and elaboration. https://doi.org/10.1136/bmj.n160.
(DOCX)
