# Supplementary material for: Plant-based therapeutics for leishmaniasis: A systematic review emphasizing human studies and clinical trial evidence
Source: PLoS Negl Trop Dis. 2026 Jun 5;20(6):e0014389. doi: 10.1371/journal.pntd.0014389 (PMC13240915; doi:10.1371/journal.pntd.0014389)
Supplement: S1 Table — (DOCX) [file pntd.0014389.s001.docx]

**Table S1.** Search strategy PubMed

| (Leishmaniasis[MeSH Terms] OR leishmania OR kala-azar OR dumdum OR cutaneous leishmaniasis OR visceral leishmaniasis OR mucocutaneous leishmaniasis OR leishmaniosis OR antileishmania*) AND (herbal medicine[MeSH Terms] OR plant-based OR plant-based compounds OR phytotherapy OR plant extracts OR medicinal plants OR plants traditional medicine OR plant*) AND (treatment outcome[MeSH Terms] OR treatment OR efficacy OR effectiveness OR cure rate OR cure OR adverse effects OR toxicity OR side effects OR clearance OR leishmanicidal effect OR IC50) |
| --- |
