# Supplementary figures and images for: Plant-based therapeutics for leishmaniasis: A systematic review emphasizing human studies and clinical trial evidence
Source: PLoS Negl Trop Dis. 2026 Jun 5;20(6):e0014389. doi: 10.1371/journal.pntd.0014389 (PMC13240915; doi:10.1371/journal.pntd.0014389)

**
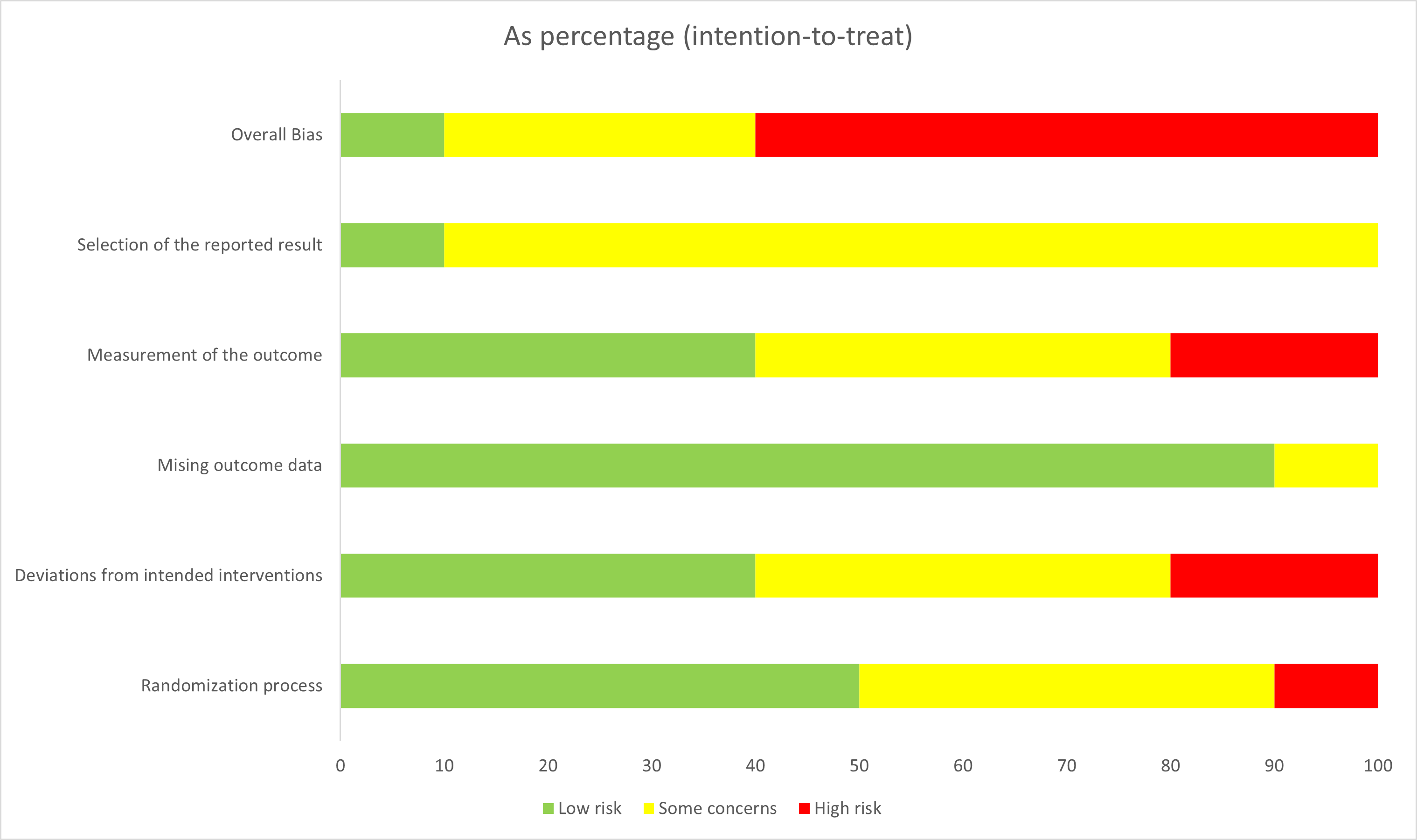
S1. Fig** The results of the risk of bias (ROB-2) assessment by the evaluated domain

Supplement: S1 Fig — (DOCX) [file pntd.0014389.s002.docx]
