# Supplementary material for: Plant-based therapeutics for leishmaniasis: A systematic review emphasizing human studies and clinical trial evidence
Source: PLoS Negl Trop Dis. 2026 Jun 5;20(6):e0014389. doi: 10.1371/journal.pntd.0014389 (PMC13240915; doi:10.1371/journal.pntd.0014389)
Supplement: S1 — Original source: Matthew J. Page et al. (2021). PRISMA 2020 explanation and elaboration. https://doi.org/10.1136/bmj.n160. (DOCX) [file pntd.0014389.s003.docx]

| **Section and Topic** | **Item #** | **Checklist item** | **Location where item is reported** |
| --- | --- | --- | --- |
| **TITLE** | | |  |
| Title | 1 | Identify the report as a systematic review. | Page 1: The title clearly identifies the report as a “systematic review.” |
| **ABSTRACT** | | |  |
| Abstract | 2 | See the PRISMA 2020 for Abstracts checklist. | The checklist is attached. |
| **INTRODUCTION** | | |  |
| Rationale | 3 | Describe the rationale for the review in the context of existing knowledge. | Page 5-6: The introduction describes the global epidemiology of leishmaniasis, its disease forms (CL, MCL, VL), the burden in affected regions, and the limitations of current chemotherapeutic options. It further discusses the economic barriers and the growing interest in plant-based therapies. The section establishes the research gap, a lack of evidence synthesis on human studies evaluating plant-based antileishmanial treatments. |
| Objectives | 4 | Provide an explicit statement of the objective(s) or question(s) the review addresses. | Page 7: Clearly stated as: “This systematic review aims to provide a comprehensive overview of the available literature on plant-based treatments used in the treatment of leishmaniasis in humans, evaluating their efficacy and safety profiles.” |
| **METHODS** | | |  |
| Eligibility criteria | 5 | Specify the inclusion and exclusion criteria for the review and how studies were grouped for the syntheses. | Page 8-10: Detailed information given on included studies and their synthesis. |
| Information sources | 6 | Specify all databases, registers, websites, organisations, reference lists and other sources searched or consulted to identify studies. Specify the date when each source was last searched or consulted. | Page 8:  PubMed, Scopus, and Web of Science were searched from inception to May 28, 2024. Reference lists of included studies were screened for additional sources. |
| Search strategy | 7 | Present the full search strategies for all databases, registers and websites, including any filters and limits used. | S1 Table of the appendix |
| Selection process | 8 | Specify the methods used to decide whether a study met the inclusion criteria of the review, including how many reviewers screened each record and each report retrieved, whether they worked independently, and if applicable, details of automation tools used in the process. | Page 8-10: Added all details, including “Two independent reviewers screened titles and abstracts.” |
| Data collection process | 9 | Specify the methods used to collect data from reports, including how many reviewers collected data from each report, whether they worked independently, any processes for obtaining or confirming data from study investigators, and if applicable, details of automation tools used in the process. | Page 9-10,  Data were independently extracted by two reviewers using a standardized form covering study design, population, intervention, comparator, and outcomes. Disagreements were resolved through discussion. |
| Data items | 10a | List and define all outcomes for which data were sought. Specify whether all results that were compatible with each outcome domain in each study were sought (e.g. for all measures, time points, analyses), and if not, the methods used to decide which results to collect. | Page 8 and 10: Highlighted the outcomes. |
|  | 10b | List and define all other variables for which data were sought (e.g. participant and intervention characteristics, funding sources). Describe any assumptions made about any missing or unclear information. | Page 9: Data extraction details described. |
| Study risk of bias assessment | 11 | Specify the methods used to assess risk of bias in the included studies, including details of the tool(s) used, how many reviewers assessed each study and whether they worked independently, and if applicable, details of automation tools used in the process. | Page 9-10: ROB-2 described. |
| Effect measures | 12 | Specify for each outcome the effect measure(s) (e.g. risk ratio, mean difference) used in the synthesis or presentation of results. | Page 10: The estimations described. |
| Synthesis methods | 13a | Describe the processes used to decide which studies were eligible for each synthesis (e.g. tabulating the study intervention characteristics and comparing against the planned groups for each synthesis (item #5)). | Page 10: In the data synthesis and analysis described. |
|  | 13b | Describe any methods required to prepare the data for presentation or synthesis, such as handling of missing summary statistics, or data conversions. | Page 10: In the data synthesis and analysis described. |
|  | 13c | Describe any methods used to tabulate or visually display results of individual studies and syntheses. | Page 10: In data synthesis and analysis described. |
|  | 13d | Describe any methods used to synthesize results and provide a rationale for the choice(s). If meta-analysis was performed, describe the model(s), method(s) to identify the presence and extent of statistical heterogeneity, and software package(s) used. | Page 10: In data synthesis and analysis described. |
|  | 13e | Describe any methods used to explore possible causes of heterogeneity among study results (e.g. subgroup analysis, meta-regression). | Not applicable |
|  | 13f | Describe any sensitivity analyses conducted to assess robustness of the synthesized results. | Not applicable |
| Reporting bias assessment | 14 | Describe any methods used to assess risk of bias due to missing results in a synthesis (arising from reporting biases). | Not applicable |
| Certainty assessment | 15 | Describe any methods used to assess certainty (or confidence) in the body of evidence for an outcome. | Not applicable |
| **RESULTS** | | |  |
| Study selection | 16a | Describe the results of the search and selection process, from the number of records identified in the search to the number of studies included in the review, ideally using a flow diagram. | Page 11-12: Text and Figure 1 |
|  | 16b | Cite studies that might appear to meet the inclusion criteria, but which were excluded, and explain why they were excluded. | Page 11: Reasons for study exclusion mentioned in the text. |
| Study characteristics | 17 | Cite each included study and present its characteristics. | Page 13-16, including Table 1. |
| Risk of bias in studies | 18 | Present assessments of risk of bias for each included study. | Page 17, including Figure 2 and Figure S1. |
| Results of individual studies | 19 | For all outcomes, present, for each study: (a) summary statistics for each group (where appropriate) and (b) an effect estimate and its precision (e.g. confidence/credible interval), ideally using structured tables or plots. | Page 20-24 including Table 2 and Figure 3. |
| Results of syntheses | 20a | For each synthesis, briefly summarise the characteristics and risk of bias among contributing studies. | Summary of risk of bias in Figure 2. |
|  | 20b | Present results of all statistical syntheses conducted. If meta-analysis was done, present for each the summary estimate and its precision (e.g. confidence/credible interval) and measures of statistical heterogeneity. If comparing groups, describe the direction of the effect. | Page 22-24 |
|  | 20c | Present results of all investigations of possible causes of heterogeneity among study results. | Not applicable |
|  | 20d | Present results of all sensitivity analyses conducted to assess the robustness of the synthesized results. | Not applicable |
| Reporting biases | 21 | Present assessments of risk of bias due to missing results (arising from reporting biases) for each synthesis assessed. | Not applicable |
| Certainty of evidence | 22 | Present assessments of certainty (or confidence) in the body of evidence for each outcome assessed. | Not applicable |
| **DISCUSSION** | | |  |
| Discussion | 23a | Provide a general interpretation of the results in the context of other evidence. | Page 23-25 |
|  | 23b | Discuss any limitations of the evidence included in the review. | Page 30-31 |
|  | 23c | Discuss any limitations of the review processes used. | Page 30-31 |
|  | 23d | Discuss implications of the results for practice, policy, and future research. | Page 31 |
| **OTHER INFORMATION** | | |  |
| Registration and protocol | 24a | Provide registration information for the review, including register name and registration number, or state that the review was not registered. | Page 9: (PROSPERO ID CRD42024567764) |
|  | 24b | Indicate where the review protocol can be accessed, or state that a protocol was not prepared. | (PROSPERO ID CRD42024567764) |
|  | 24c | Describe and explain any amendments to information provided at registration or in the protocol. | No amendments were made |
| Support | 25 | Describe sources of financial or non-financial support for the review, and the role of the funders or sponsors in the review. | Page 31 |
| Competing interests | 26 | Declare any competing interests of review authors. | Page 31: The authors have declared that no competing interests exist |
| Availability of data, code and other materials | 27 | Report which of the following are publicly available and where they can be found: template data collection forms; data extracted from included studies; data used for all analyses; analytic code; any other materials used in the review. | Page 32 |

*From:*  Page MJ, McKenzie JE, Bossuyt PM, Boutron I, Hoffmann TC, Mulrow CD, et al. The PRISMA 2020 statement: an updated guideline for reporting systematic reviews. BMJ 2021;372:n71. doi: 10.1136/bmj.n71. This work is licensed under CC BY 4.0. To view a copy of this license, visit <https://creativecommons.org/licenses/by/4.0/>
